# Letter on: “An analysis of deficiencies in the data of interventional drug trials registered with Clinical Trials Registry – India”

**DOI:** 10.1186/s13063-019-4010-3

**Published:** 2020-01-07

**Authors:** Mohua Maulik, Jyotsna Gupta, Atul Juneja, Tulsi Adhikari, Saurabh Sharma, Yashmin Panchal, Neha Yadav, Mendu Vishnu Vardhana Rao

**Affiliations:** 1 Clinical Trials Registry - India, ICMR-National Institute of Medical Statistics, New Delhi, Delhi 110029 India; 2 ICMR-National Institute of Medical Statistics, New Delhi, Delhi 110029 India

**Keywords:** Clinical Trials Registry, India, CTRI, Automated analysis

## Abstract

An article published in this journal analyses the deficiencies in the data of interventional drug trials registered with Clinical Trials Registry - India. We wish to rebut some of the inferences and highlight the pitfalls of a purely automated analysis of registry data as posited by the authors.

## Main Text

Pillamarapu et al. [1] reported on the various categories of problems with the data in the Clinical Trials Registry – India (CTRI) database, including (1) a lack of clarity in the classification of the types of study, (2) internal inconsistencies, (3) incomplete or non-standard information, (4) missing data, (5) variations in names or classification and (6) incomplete or incorrect details on the ethics committees.

The effort and the detailed scrutiny of the CTRI data are much appreciated, and many suggestions, particularly the use of logic to reduce internal inconsistencies, are very useful. Drop-down lists are indeed an effective way for reducing the inconsistencies of data entry. In fact, several mandatory drop-down lists were incorporated into the CTRI software in 2011 and more recently in 2018, whereupon the health condition field was coded as per the International Classification of Diseases, Tenth Revision (ICD-10). We look forward to also learning from this article and, where feasible, implementing more drop-down lists to capture more standard information that is amenable to automated analysis. However, we submit that the application of only automated analysis on registered trial data is likely to lead to errors in interpretation.

For instance, for the quoted 1% (22) of the trials which were analysed as foreign trials, as per our data (Table 1) only two trials are “true foreign trials”. Of the remaining 20 trials, five trials are Indian trials terminated post-registration, while in six trials, India has been removed as a participating country post-registration, and this information is available in the public domain as part of the audit trail. The registrants of these trials have been requested to provide the status of the trial in India in the brief summary section. For the remaining nine trials, one trial registrant had been asked twice before trial registration to update the country of recruitment but did not respond. There was true oversight in eight trials and not in 22 trials as reported. Hence, we feel that for a registry like CTRI, purely automated analysis is perhaps not a viable option.

**Table 1:** Account of errors observed in 22 foreign trials

CTRI number	Error account
CTRI/2011/07/001877	CTRI oversight
CTRI/2007/091/000042	*India removed post-registration
CTRI/2012/02/002443	Terminated trial in India
CTRI/2009/091/000240	*India removed post-registration
CTRI/2011/10/002050	Terminated trial in India
CTRI/2011/07/001898	Request sent twice prior to registration but not heeded
CTRI/2011/07/001867	*India removed post-registration
CTRI/2011/09/002020	CTRI oversight
CTRI/2010/091/001403	*India removed post-registration
CTRI/2011/09/001983	CTRI oversight
CTRI/2010/091/006103	*India removed post-registration
CTRI/2011/11/002126	CTRI oversight
CTRI/2011/08/001973	CTRI oversight
CTRI/2012/04/002555	Terminated trial in India
CTRI/2012/10/003082	CTRI oversight
CTRI/2014/01/004298	*India removed post-registration
CTRI/2015/03/005617	CTRI oversight
CTRI/2016/05/006952	*India removed post-registration
CTRI/2017/08/009558	CTRI oversight
CTRI/2010/091/001049	Terminated trial in India
CTRI/2015/12/006458	True foreign trial
CTRI/2017/06/008736	True foreign trial

* Information available in the public domain under “modifications” link in relevant field

Similarly, most of the reported “deficiencies” in the article are also likely because of inadequate understanding and misconceptions regarding the CTRI data, some of which are highlighted below.

With regard to ethics committee details, once all ethics committees are registered, either with the Central Drugs Standard Control Organization or the Department of Health Research as per the New Drugs and Clinical Rules 2019 (https://cdsco.gov.in/opencms/export/sites/CDSCO_WEB/Pdf-documents/NewDrugs_CTRules_2019.pdf), linkage to those registrations and validation of the ethics committees can be attempted by the CTRI platform for the convenience of all stakeholders. However, a mismatch in the number of sites with ethics approval can be observed because the number of sites in a multicentre trial is a dynamic field, with sites being added and removed over the course of a study. In this context, registrants must first upload ethics approvals of new sites before the site field is manually unlocked for inclusion of new sites in an effort to ensure site data are kept up to date in the registry. This process is likely to have contributed to the error rates quoted by the authors.

With regard to missing data, we would like to reiterate that the CTRI software was revised in March 2011, whereupon several new drop-down lists were included. Hence, trials registered prior (1650 registrations) to this revision had missing data for several fields. Registrants were repeatedly requested to update the data fields as per the revised dataset form, and to date, approximately 604 trials still have not been updated, although the exact number may vary for an individual field. CTRI itself has no access to any data field, nor does it have any regulatory authority or powers. For any change in a field (even for an obvious error), the CTRI can only send a request (not enforce) to the registrant to update the records.

Regarding principal investigator fields, the authors have noted that 5% of Indian trials and 40% of global trials did not report PI details. As in ClinicalTrials.gov, this field is not a compulsory field in the CTRI because it is over and above the dataset items specified by the WHO and, hence, should not be counted as an error.

We also would like to highlight some of the numerical errors in the article such as there currently being 17 primary registries and not the 18 mentioned. Furthermore, considerable discrepancy, as well as inconsistency, exists in the number of registered trials reported for the CTRI.

As the exact dates for 2008, 2009 and 2015 are not quoted, we presumed that these refer to data until 31 December for the particular year. However, the quoted reference [2] mentions the specific date for 29 trials (31 March 2008) and 155 trials (10 January 2009). As shown in Table 2, where exact dates are quoted, the number obtained from CTRI are also incorrect. Although issues occur when searching and downloading CTRI data in the public domain, the display of registered trials number has been checked and verified to match that obtained by database search. Furthermore, the methodology of the “error rates” provided in the additional files are quite incomprehensible.

**Table 2:** Comparative registration numbers

Date	Reported	As per CTRI records
2008	29	141
2009	155	686
2015	6474	6474
30/06/17	8969	8949
04/04/18	12,673	13,049
25/06/19	19,830	19,955

At the CTRI, we are of the opinion that the title of the article “An analysis of deficiencies in the data of interventional drug trials registered with Clinical Trials Registry - India” is rather misleading and unnecessarily sensational, as hidden inside the depths of the article is the statement “For the majority of problems that we have quantified, the error rates are in single digits. This is creditable.” This statement is contradictory to the implications of the title and the abstract, which is what is read by most readers.

Interestingly, the authors have also published an article on “Some data quality issues at ClinicalTrials.gov” [3], where they have found missing data and variation in names as well “junk” information in the PI field to the tune of 35% of ClinicalTrials.gov records. However, despite meticulous comparisons to ClinicalTrials.gov, no mention is made of the proportion of such junk data in the CTRI. If no junk data are present in CTRI (as is our contention), a mention of that fact would have made this a more balanced and impartial article.

## Data Availability

Not applicable.

## Abbreviations

CTRI: Clinical Trials Registry – India
ICD-10: International Classification of Diseases, Tenth Revision
PI: principal investigator
